# Pattern of Drug Use and Depressive Symptoms among Amphetamine Type Stimulants Users in Beijing and Guangdong Province, China

**DOI:** 10.1371/journal.pone.0060544

**Published:** 2013-04-09

**Authors:** Yan-ping Bao, Yi Qiu, Shi-yan Yan, Zhen-jun Jia, Su-xia Li, Zhi Lian, Yue Mu, Zhi-min Liu

**Affiliations:** 1 National Institute on Drug Dependence, Peking University, Beijing, China; 2 Institute of Basic Research in Clinical Medicine, China Academy of Chinese Medical Sciences, Beijing, China; Peking University, China

## Abstract

**Background:**

In recent years, amphetamine-type stimulants (ATS) have increased dramatically in East-south Asia, especially in China. Most ATS users suffered from psychosis comorbidity, and depression is the main syndrome in ATS users.

**Methodology:**

A cross-sectional study of depressive symptoms and associated factors among ATS users was conducted in compulsory and voluntary drug detoxification and rehabilitation centers of Beijing and Guangdong Province from March, 2010 to August, 2010. Total 402 eligible participants were recruited and investigated by trained interviewers using a structured questionnaire, the depression was measured by the short 13-item Beck Depression Inventory (BDI-13). Multiple logistic regression was used to determine the impact of associated risk factors of depressive symptoms (%≥8).

**Principle Finding:**

The mean score of BDI-13 is 8.11, and 169 participants (42.04%) have depressive symptoms, including 106 (26.37%) with moderate and 63 (15.67%) with severe depressive symptoms. Higher dose of ATS use, history of ATS relapse were associated with moderate and severe depressive symptoms, the adjusted odds ratios (OR) was 2.62, (95% CI: 1.45–4.74) and 2.01 (95% CI: 1.18–3.42) respectively. The cessation of 12 months or more had less risk of depressive symptoms than the current users, the OR was 0.46 (95% CI: 0.24–0.91), and the ATS users reporting nicotine dependence and alcohol drinking had significantly more risk of depressive symptoms for 3.11 (1.83–5.28) and 2.22 (1.35–3.65) times than those without these behaviors.

**Conclusions:**

Depressive symptoms co-occurred frequently among ATS users in China. The efforts that facilitate drug users’ attempts to stop using ATS use and relapse, quit cigarette smoking and stop alcohol drinking during the ATS treatment and management process should be supported as they may contribute to improving the mental health among this population.

## Introduction

The use of amphetamine-type stimulants (ATS) is recognized as a major global public health problem [1]. The 2012 World Drug Report showed that the ATS is the second leading substance of abuse following cannabis worldwide, with estimates that up to 52 million individuals (1.2% of the global population aged 15–64 years) have used ATS at least once in the past 12 months [2]. East-south Asia have experienced significant increase of ATS use, particularly among young people [3]. In China, the main element of new synthetic drugs emerged recent years is ATS which including methamphetamine (MA), methylenedioxymethamphetamine (MDMA), and so-called “Magu” pills (which are typically capsules containing a mixture of MA and other drugs such as caffeine) [4], [5]. Data from the China National Narcotic Control Commission showed that 59,500 people used or abused synthetic drug in 2005, accounting for 6.7% of registered drug users [6]. However, by the end of 2011, this number had swelled to an estimated 0.59 million people (32.7% of the 1.79 million registered drug users) and among them 67.8% were under 35 year’s young people [5].

Depression often co-occurs with amphetamine type stimulants use and the link between ATS use and depression has been reported in previous studies [4], [7], [8]. Our early studies showed that the most ATS users had suffered from psychosis comorbidity, and one of the main syndromes was depression [4]. An Australia study showed that 79% methamphetamine users reported history of depression, and an American epidemiological data showed 41.6% of adults with amphetamine use disorders have a lifetime history of depression [9], [10]. Recent years, some studies focus on the temporal relationship between depression and ATS use, a prospective cohort study from Australia indicated that ATS initiation during adolescence was associated with adulthood depression, whereas early depression was not predictive of future ATS use [11]. Some studies showed that the patients seeking treatment for ATS dependence are more likely to be diagnosed with depression [12]. The factors related drug use were reported to be associated with depression, such as early onset ATS use (initiation use at 15 years or younger), the administration of injection and more frequent (≥4 days per week) ATS use [7], [13], [14]. Depression have controversial association with cessation of ATS use, a study indicated that depression is a major component of the withdrawal syndrome of ATS use during the first 3 weeks of abstinence [15], however an prospective cohort study found that depressive symptoms decreased significantly among those who stopped using ATS over the 12-month study period [8].

With the dramatically increase of ATS users in China, the public health burden of psychiatric co-morbidities associated with ATS use may have increased. However, little is known about the status and associations of depression among ATS users in China. This study aims to find the relation between pattern of ATS use and the depressive symptoms and to examine its potential associated factors among this population in China. Such findings would provide information for the treatment and management strategies for ATS use and associated mental health in this population.

## Materials and Methods

### Ethics Statement

This research protocol was approved by the Institutional Review Board of Peking University Health Center and written informed consent was obtained from all study participants. At each study site, the trained interviewers explained the meaning and aim of the survey to the potential participants and let them to know that this is a scientific study which would keep secret information for all participants before the investigation in a separate room. Because the participants in compulsory drug detoxification center belong to a traditionally vulnerable group, the interviewers explained the detailed process of investigation and make sure that all potential participants who declined to participate or otherwise did not participate were not disadvantaged in any other way by not participating in the study. If the potential participants did not clearly understand, interviewers explained again. After they understand the study completely and signed informed consent, the face-to-face interviews were conducted by trained interviewers using a self-administered structured questionnaire that included demographic characteristics, drug use history, general information of cigarette smoking, drinking behavior and depression.

### Participants and Setting

A cross-sectional study of depression and risk behavior among ATS users was conducted in compulsory and voluntary drug detoxification and rehabilitation centers in Beijing and Guangdong Province from March 2010 to August 2010. The study design used convenience sampling to recruit any patients who meet study inclusion criteria: (1) at least 18 years old, (2) urine test positive for at least one of ATS drug, including MA, “Magu” pills (the main component of which is MA), and MDMA, at the time they entered the detoxification center, and (3) meeting the Diagnostic and Statistical Manual of Mental Disorders (DSM-IV; American Psychiatric Association) criteria for ATS abuse or dependence, and (4) signed informed consent. The participants were excluded if they had serious physical illnesses such as an acute stage of cardiovascular disease, cerebrovascular disease, or neurodegenerative diseases.

### Measures and Test

At each study site, the trained interviewers explained the meaning and aim of the survey to the potential participants and let them to know that this is a scientific study which would keep secret information for all participants before the investigation in a separate room. Because the participants in compulsory drug detoxification center belong to a traditionally vulnerable group, the interviewers explained the detailed process of investigation and make sure that all potential participants who declined to participate or otherwise did not participate were not disadvantaged in any other way by not participating in the study. If the potential participants did not clearly understand, interviewers explained again. After they understand the study completely and signed informed consent, the face-to-face interviews were conducted by trained interviewers using a self-administered structured questionnaire that included demographic characteristics, drug use history, general information of cigarette smoking, drinking behavior and depression. The demographic variables included gender, age, ethnicity, education, marital status, and employment. Questions about drug use history investigated the types of ATS ever used and the main type of drug use, frequency, duration, and administration of ATS use. Cigarette smoking and nicotine dependence were collected by the Fagerström Test for Nicotine Dependence (FTND) [16]. Symptoms of depression were measured using the short 13-item Beck Depression Inventory (BDI-13). Each item is scored from 0 to 3 with a maximum score of 39. The score of 0–4, 5–7, 8–15, ≥16 were classified as no depression, mild, moderate and severe depression respectively [17], [18]. Participants self-administered the BDI under the supervision of a research staff who read the questions aloud for those who requested help or had difficulty reading during interview.

### Statistical Analysis

The data were double entered, and the consistency of both databases was compared using Epi Data software (Epi Data Association, Odense, Denmark). The estimations of the means and proportions were calculated to describe the demographic characteristics, drug use history, cigarette smoking, nicotine dependence, alcohol drinking, BDI score and prevalence of depressive symptoms. Pearson chi-square test and bivariate logistic regression were used for the analysis of demographic characteristics, drug use history, cigarette smoking and alcohol drinking with different depression status. A multiple logistic regression model was constructed using a stepwise backward sequence. All variables that were significantly associated with the endpoint based on the univariate logistic regression were then entered in a multiple logistic regression model to adjust for all other variables. Potential confounding effects and effect modification were examined for: current age, education level, occupation etc. All analyses were performed using SAS software, version 9.1 (SAS Institute, Cary, NC). Statistically significant findings were determined using a 2-tailed P value of 0.05.

## Results

### Sample Characteristics and Pattern of Drug Use

Among 402 eligible participants, 202 participants were recruited from the compulsory drug detoxification and rehabilitation center in Beijing, 121 and 79 participants were recruited from the compulsory and voluntary drug detoxification center respectively in Guangdong Province. Demographic and drug-use characteristics of participants are summarized in Table 1. Participants were typical of ATS users in China, with a predominance of single unemployed young males. The ages of the participants ranged from 18 to 52 years (mean, 31.29 years) and 269 participants (66.92%) were young people aged less than 35 years. The majority (94.50%) belonged to the Han ethnic group, 302 (75.12%) were male, 282 (71.39%) completed primary school or junior high school, 156 (39.20%) were unemployed, and 204 (50.75%) were unmarried.

**Table 1:** Demographic characteristics and drug use history of 402 ATS Users.

	Beijing	Guangdong	Total
Variables	(n = 202) n (%)	(n = 200) n (%)	(n = 402) *n (%)*
Age (Years, Mean±Sd)*	32.727.75	29.787.14	31.29±7.52
Age (<35 years)*	124 (61.39)	145 (72.50)	269 (66.92)
Compulsory of detoxification center*	202 (100.00)	121 (60.50)	323 (80.35)
Ethnicity-Han*	182 (90.55)	179 (98.90)	361 (94.50)
Male gender	144 (71.29)	158 (79.00)	302 (75.12)
Junior high school or less	138 (69.70)	144 (73.10)	282 (71.39)
Unemployed	83 (41.50)	73 (36.87)	156 (39.20)
Unmarried *	95 (47.03)	109 (54.50)	204 (50.75)
Types of ATS mainly used*			
Methamphetamine	194 (96.04)	186 (93.00)	380 (94.53)
Ecstasy	4 (1.98)	0 (0)	4 (1.00)
Magu pill	4 (1.98)	14 (7.00)	18 (4.48)
Administration of drug use			
Inhalation administration	197 (97.52)	195 (97.50)	392 (97.51)
Poly drug use*	112 (55.45)	79 (39.70)	191 (47.51)
History of heroin use*	59 (29.21)	15 (7.54)	74 (18.45)
Duration of ATS ever used ≤2 months*	60 (30.61)	21 (10.71)	81 (20.66)
Frequency of ATS use (Each day)	106 (52.48)	71 (35.50)	177 (44.03)
Dose of ATS use >0.3g/day*	164 (81.19)	117 (58.50)	281 (69.90)
Cessation 12 month or more	10 (4.95)	180 (90.00)	190 (47.26)
Cause of drug use of first time* #			
Curious	98 (48.51)	129 (64.50)	227 (56.47)
influence by friends or peers	85 (42.08)	105 (52.50)	190 (47.26)
Pursuit of euphoria	34 (16.83)	92 (46.00)	126 (31.34)
Alleviating distress or unpleasant emotions	24 (11.88)	31 (15.50)	55 (13.68)
Get energy	27 (13.37)	16 (8.00)	43 (10.70)
To socialize	25 (12.38)	2 (1.00)	26 (6.47)
Source of drug* #			
Illegal drug market	95 (47.50)	170 (85.00)	265 (66.25)
Peers or friends	81 (40.50)	41 (20.50)	122 (30.50)
Entertainment places	37 (18.50)	55 (27.50)	92 (23.00)
The person of use together* #			
Friends	113 (55.94)	142 (71.00)	255 (63.43)
Girl/boy friend	78 (38.61)	48 (24.00)	126 (31.34)
By himself	56 (27.72)	30 (15.00)	86 (21.39)
Waiters in entertainment place	14 (6.93)	26 (13.00)	40 (9.95)
Alcohol drinking	100 (52.63)	99 (49.75)	208 (51.4)
Cigarette smoking	194 (96.52)	194 (97.00)	388 (96.76)
FTND score (Years, Mean±Sd)*	4.77±2.41	5.60±2.89	5.2±2.7
Severe nicotine dependence (FTND ≥7)*	44 (23.53)	76 (40.21)	124 (32.2)

* there was significant difference between Beijing and Guangdong, p<0.05.

# multiple responses.

Among the 402 ATS users, methamphetamine was the main drug used in the participants and the majority (97.51%) used ATS by inhalation administration. One hundred ninety one participants (47.51%) had a history of polydrug use, indicating the use of 2 or more drugs, including methamphetamine, ecstasy, “magu” pills, ketamine, heroin and other opiates, tramadol, cocaine, cannabis, and benzodiazepine. Curious (56.47%), influence by friends or peers (47.26%), and pursuit of euphoria (31.34%) were the main causes at the first time of drug use. Illegal market (66.25%), peers or friends supply (30.50%) were the main sources of drugs, and about 23% drugs were from entertainment area. Most of ATS users (63.43%) used drug together with friends or peers, less 10% used ATS with strange waiters in entertainment areas. About 51.4% and 96.76% ATS users had behavior of alcohol drinking and cigarette smoking during the period of ATS use.

The drug users of Beijing and Guangdong Province had different special characteristics, the important features of ATS users in Beijing included high dose of ATS use, more length duration of ATS use and more proportion of poly drug use, especially more heroin abuse history than those in Guangdong. Compared with Beijing, the ATS users from Guangdong province where had more ATS Manufacturing plant had some features, including younger, lower dose of ATS use each time, less poly-drug use history, more curious and peer influence, more drugs from entertainment area (Table 1).

### The Depressive Symptoms and Associated Factors among ATS Users

According to BDI score, 169 participants (42.04%) had depressive symptoms, including 106 (26.37%) with moderate and 63 (15.67%) with severe depressive symptoms. Table 2 listed the factors which were associated with moderate or severe depressive symptoms (BDI-13 score ≥8) by univariate logistic regression analysis, including age, gender, area, occupation, duration of ATS ever used, cessation of drug use, dose of ATS use, relapse history, using ATS together with entertainment venues waiter, sleep disorder, hypomnesia and promiscuity after using ATS, cigarette smoking, nicotine dependence and alcohol drinking. The multiple logistic regression showed that the high dose for ATS use, history of ATS use relapse were associated with depressive symptoms, the adjusted odds ratios (OR) was 2.62, (95% CI: 1.45–4.74) and 2.01 (95% CI: 1.18–3.42) respectively. The cessation 12 month or more have less risk of depressive symptoms than the current users, the OR was 0.46 (95% CI: 0.24–0.91), and the ATS users reporting nicotine dependence and alcohol drinking were significantly have more risk of depressive symptoms for 3.11 (1.83–5.28) and 2.22 (1.35–3.65) times than those without these behaviors (Table 3).

**Table 2:** Bivariate analysis of risk factors for depressive symptoms among ATS Users.

		Depression (BDI-13 score≥8)			
Characteristics	*N*	n	%	OR	95%CI#
Age (years)					
≤34	269	102	37.92	1.0	
≥35	133	67	50.38	1.66	1.09–2.53*
Gender					
Female	100	20	20.00	1.0	
Male	302	149	49.34	3.87	2.26–6.64*
Area					
Beijing	202	71	35.15	1	
Guangdong	200	98	49.00	1.77	1.19–2.65*
Type of drug detoxification center					
Voluntary	79	39	49.37	1	
Compulsory	323	130	40.25	0.69	0.42–1.13
Nationality					
Han	361	155	42.94	1.0	
Minority	21	8	38.10	0.82	0.33–2.02
Marital status					
Unmarried	204	77	37.75	1.0	
Cohabitating	17	7	41.18	1.13	0.55–2.30
Married	144	70	48.61	1.56	1.01–2.40
Divorced or widowed	37	15	40.54	1.16	0.42–3.16
Education					
Junior high school or less	282	124	43.97	1.0	
Senior high school or more	113	41	36.28	0.73	0.46–1.14
Occupation					
Unemployed	156	42	26.92	1.0	
Employed	242	125	51.65	2.90	1.88–4.48*
Duration of ATS ever used (months)					
0–2	81	26	32.10	1.0	
≥3	311	138	44.37	1.69	1.01–2.83*
Period of drug use					
Continue use	190	91	47.89	1.0	
Cessation 1–12 month	131	54	41.22	1.67	0.92–3.01
Cessation 12 month or more	81	24	29.63	2.18	1.25–3.80*
Dose of ATS use (g/day)					
≤0.3	121	31	25.62	1.0	
>0.3	281	138	49.11	2.80	1.75–4.49*
History of relapse					
No	264	98	37.12	1.0	
Yes	137	71	51.82	1.82	1.20–2.77*
Polydrug use					
No	210	83	39.52	1.0	
Yes	191	86	45.03	1.25	0.84–1.86
Use ATS together with entertainment venues waiter					
No	362	145	40.06	1.0	
Yes	40	24	60.00	2.25	1.15–4.37*
Sleep disorders frequency after using ATS					
Never or occasionally	150	43	28.67	1.0	
Each time	214	98	45.79	2.10	1.35–3.28*
Hypomnesia after using ATS					
Never	112	24	21.43	1.0	
Mild	170	70	41.18	2.57	1.49–4.43*
Severe	115	74	64.35	6.82	3.67–11.95*
History of promiscuity after use ATS					
No	345	135	39.13	1.0	
Yes	57	34	59.65	2.30	1.30–4.07*
Cigarette smoking					
No	17	2	11.76	1.0	
Yes	364	157	43.13	5.69	1.28–25.24*
The change of number of cigarette smoking after drug taking					
No change	167	52	31.14	1.0	
Decrease	32	16	50.00	2.21	1.03–4.76*
Increase	177	96	54.24	2.62	1.69–4.07*
Nicotine dependence (FTND score)					
<7	256	83	32.42	1.0	
≥7	120	79	65.83	4.02	2.54–6.36*
Alcohol Drinking					
No	190	64	33.68	1.0	
Yes	199	102	51.26	2.07	1.37–3.12*

* : there was significant difference, p<0.05.

# :95% confidential interval (95%CI).

**Table 3:** Multiple Logistic regression of risk factors for depressive symptoms among ATS Users.

Characteristics	Adjusted OR for (BDI-13 score≥8)	95%CI#
Dose of ATS use (g/day)		
≤0.3	1.0	
>0.3	2.62	1.45–4.74*
History of relapse		
No	1	
Yes	2.01	1.18–3.42*
Period of drug use		
Continue use	1.0	
Cessation 1–12 month	0.57	0.32–1.04
Cessation 12 month or more	0.46	0.24–0.91*
Nicotine dependence (FTND score)		
<7	1.0	
≥7	3.11	1.83–5.28*
Alcohol Drinking		
No	1.0	
Yes	2.22	1.35–3.65*

* there was significant difference, p<0.05.

# 95% confidential interval (95%CI).

## Discussion

This cross-sectional study found some characteristics of drug use and reported a high rate (42%) of depressive symptoms among ATS users in Beijing and Guangdong Province, China. We showed additional information about the factors associated with depressive symptoms in ATS users. These findings provide evidence of the development strategies for prevention and treatment of ATS use and associated depressive symptoms in this population.

In this study, we found some features of ATS users in Guangdong Province, where is the south-eastern littoral and rich province and close to Hong Kong and Macao. Resent years, a large number of new ATS users appears in Guangdong Province, where became one of the provinces with top numbers of registered ATS users [3]. The findings indicated that the ATS users in Guangdong Province were younger and vulnerable by peers and friends than those in Beijing, which suggested that more attention should be paid to health education about ATS use prevention in the areas of ATS abuse spread including Guangdong Province.

Depressive symptoms were common in ATS users and the rate of moderate or severe depression was high (42%). This finding which the co-occurrence of high level of depressive symptoms was common in ATS users, was approved by studies from China and other countries [19], [20]. Some studies showed that the prevalence of depression ranged from 28%-80% among ATS users or ATS dependent patients seeking treatment [9], [10], [19], [20]. This study supports the findings of these authors and substantially broadens the reference population.

The multiple logistic regression analysis showed that high dose of ATS use, history of relapse, current ATS use, nicotine dependence and alcohol drinking were associated with moderate or severe depressive symptoms. The study showed that ATS users with high dose of drug use, which was related with higher frequency of drug use, have high risk of depression. The familiar finding was observed in a Thailand study that indicated frequent methamphetamine use was associated with depressive symptoms [14]. This study indicated that ATS users of cessation 12 month or longer period had significantly less risk of depressive symptoms than participants who continued to use ATS and a similar finding was observed from a prospective cohort study [8]. The ATS users with a history of relapse have more depressive symptoms, because the history of relapse was associated with more severe degree of ATS dependence. These findings provide evidence for that the cessation and reduction of ATS use and relapse prevention were helpful to decrease the burden of ATS use and related mental health in this population.

Many studies showed that the depression and smoking or drinking have complicated bidirectional relationship [21], [22]. Research showed that cigarette smoking were significantly related to later development of major depression and other mental disorders, and inversely, depressed smoker less likely to have quit compared with non-depressed smokers [23], [24]. Studies showed that depressive symptoms were positively associated with one year alcohol use among adolescent [21]. In this study, we found that nicotine dependence and alcohol drinking during period of drug use increased and aggravated the degree of depression among ATS users. The findings indicated that reduction and treatment of nicotine dependence and alcohol drinking during ATS treatment may improve the mental health among the ATS users.

This survey showed that ATS users receiving more education have less likely to depressive symptoms indicated that the health education is helpful to decrease the ATS use and improve the mental health among ATS users. In this study, the majority participants were from compulsory drug detoxification center, we want to know whether the depression status was influenced by type of drug detoxification center. The finding that there was no difference of depressive symptoms among ATS users between compulsory and voluntary drug detoxification centers in this analysis, indicated that the method of recruitment sample had not effect on the result of depression status among ATS users in this paper. The result, the rate of depressive symptoms in male was higher than that of female in the univariate analysis, was bewildering and the result was not consistent with other study [19]. The possible reason is the female had less dose (0.29g/time) of drug use than that in male (0.39g/time) in this sample. However, the gender factor did not enter the final model in multiple logistic regression. So the gender difference of depressive symptoms among ATS users needs further investigation with rigorous design.

There were several limitations of cross-sectional investigation and non-random sampling methods in this study which would cause the difficulty in extrapolating the results. First, our samples were recruited from compulsory and voluntary drug detoxification and rehabilitation centers and should not be considered representative of all ATS users in these areas. On the other hand, this is a retrospective study and cannot avoid recall bias for the drug use history. Third, the proportion of current ATS use between the sample of Beijing and Guangdong is different, which have influence on the relationship between factors and depressive symptoms. Finally, the small sample size and limited geographic coverage might limit the detection of potentially associated risk factors. More well-designed community-based epidemiologic studies with larger sample sizes and larger geographic coverage should be conducted to validate the prevalence rate of depressive symptoms and explore the extensive risk factors using other recruitment methods.

## Conclusion

The findings indicated a higher prevalence of depressive symptoms among ATS users, and identified possible factors associated with depressive symptoms, including current ATS use, higher dose of ATS use, history of relapse, nicotine dependence, and alcohol drinking. Our study provides information for the treatment and management strategies for ATS use and associated mental health in this population for the future.
